# Fundus autofluorescence lifetimes in age‐related macular degeneration versus healthy controls in a pseudophakic population

**DOI:** 10.1111/aos.17519

**Published:** 2025-05-14

**Authors:** Jana Oertel, Dominik Fischer, Melih Tarhan, Daniel Meller, Martin Hammer

**Affiliations:** ^1^ Department of Ophthalmology University Hospital Jena Jena Germany; ^2^ Center for Medical Optics and Photonics University of Jena Jena Germany

**Keywords:** age‐related macular degeneration, fluorescence lifetime, fundus autofluorescence, lens fluorescence

## Abstract

**Purpose:**

To check whether prolonged fundus autofluorescence (FAF) lifetimes in age‐related macular degeneration (AMD) could be an artefact resulting from lens fluorescence.

**Methods:**

Fluorescence lifetime imaging ophthalmoscopy (FLIO) was performed in pseudophakic intermediate AMD as well as healthy controls. The median values of FAF lifetimes in the centre, the inner and the outer ring of the ETDRS grid, obtained as amplitude‐weighted mean of the lifetimes from a three‐exponential fit of the fluorescence decay over time in two spectral channels, as well as peak emission wavelengths (PEW) were compared between patients and controls. The age dependence of FAF lifetime was checked per group. In the patient cohort, FAF lifetimes of individuals with and without subretinal drusenoid deposits (SDD) were compared.

**Results:**

Forty‐four AMD patients (mean age 80.0 ± 6.0 years) and 26 controls (mean age 73.0 ± 10.2 years) were included. The FAF lifetimes of a subgroup of patients (*N* = 25, mean age 76.3 ± 5.6 years), age‐matched to the controls, were significantly longer than those of the controls (all grid areas and spectral channels *p* < 0.001). FAF lifetimes increased with age in the controls (*p* = 0.006–0.03), but not in the patients. Patients with SDD had longer FAF lifetimes than those without (*p* = 0.003–0.068). PEW neither showed significant group differences nor age dependence.

**Conclusions:**

Although long fluorescence lifetimes of the lens can affect FAF lifetime measurements, prolonged FAF lifetimes in AMD are specific to the disease and not a lens artefact as shown in pseudophakic eyes. The effect of AMD on the lifetimes outweighs that of age. Patients with SDD, who have a higher risk of AMD progression, also show longer FAF lifetimes.

## 1 INTRODUCTION

While fundus autofluorescence (FAF) imaging gives a qualitative impression of fluorescence intensities, fluorescence lifetime imaging ophthalmoscopy (FLIO) characterizes fluorophores, along with their embedding matrix, by their fluorescence lifetime (Schweitzer et al., 2007). This is the time a molecule remains in an excited electronic state after short pulse laser excitation. FLIO has been used to study a variety of diseases, including diabetic retinopathy (DR) (Schweitzer et al., 2015) and age‐related macular degeneration (AMD) (Dysli et al., 2016, 2017; Goerdt et al., 2021; Hammer et al., 2020; Sauer et al., 2019; Sauer, Andersen, et al., 2018; Sauer, Gensure, et al., 2018; Schultz, Hasan, Curcio, et al., 2021; Schultz, Hasan, Schwanengel, et al., 2021; Schultz, Klemm, et al., 2021; Schultz, Schwanengel, et al., 2021; Simon, Brauer, et al., 2022; Simon, Curcio, et al., 2022). As AMD shows a specific FAF lifetime pattern already in very early stages of the disease (Sauer, Andersen, et al., 2018; Sauer, Gensure, et al., 2018) and prolongation of FAF lifetimes can highlight pathologic changes of the retinal pigment epithelium (RPE), which are associated with disease progression in the follow‐up of patients (Schultz, Hasan, Curcio, et al., 2021; Schultz, Hasan, Schwanengel, et al., 2021; Schultz, Klemm, et al., 2021; Schultz, Schwanengel, et al., 2021; Schwanengel et al., 2022; Weber et al., 2022), FLIO can contribute to the diagnostics and risk management of AMD patients. Whereas patterns of lifetimes (Sauer, Andersen, et al., 2018; Sauer, Gensure, et al., 2018) and local differences (Hammer et al., 2021; Schwanengel et al., 2022; Weber et al., 2022) can be well studied, the absolute lifetime values may be skewed by the fluorescence of the ocular lens. This specifically applies to elderly subjects and cataract lenses (Brauer et al., 2020; Schweitzer et al., 2020).

FLIO uses a confocal scanning technique. This suppresses all out‐of‐focus light. Therefore, lens fluorescence is also suppressed. However, the lens is a highly fluorescent tissue (Brauer et al., 2020). This fluorescence results from tryptophan, its oxidation products (Gakamsky et al., 2017) and insoluble protein fractions (Bessems et al., 1987), with the fluorescence maxima at 515 nm (Zuclich et al., 2005). With age, lens fluorescence increases due to the accumulation of 3‐hydroxykynurenine glucoside (Van Heyningen, 1971), 4‐(2‐amino‐3‐hydroxyphenyl)‐4‐oxobutanoic acid (Truscott et al., 1994) and glutathione‐3‐hydroxykynurenine glycoside (Bron et al., 2000). Thus, lens fluorescence can overlay that of the fundus despite confocal imaging. The fluorescence lifetime of the lens is considerably longer than that of the fundus (Schweitzer et al., 2007). This might question earlier findings of prolonged FAF lifetimes in patients with AMD. In this study, we investigated whether there is an increase in lifetime due to retinal or RPE pathology in AMD, or whether the apparent increase results from the contribution of long‐living lens fluorescence. Therefore, we compared FAF lifetimes in cohorts of pseudophakic AMD patients and healthy controls. As the fluorescence of artificial intraocular lenses is negligible (Brauer et al., 2020), this provides us with true FAF lifetimes, unaffected by lens fluorescence.

## 2 METHODS

### 2.1 Subjects and procedures

Pseudophakic patients with early or intermediate AMD and pseudophakic healthy controls were recruited from the outpatient clinic of the University Hospital Jena, Department of Ophthalmology. Patients with late‐stage AMD (complete RPE and outer retinal atrophy in optical coherence tomography (OCT), macular neovascularization; Spaide et al., 2020) or diabetic retinopathy were excluded from the study. Additional exclusion criteria were a history of ocular surgery other than uncomplicated cataract extraction and YAG‐laser capsulotomy, as well as conditions such as vascular occlusion, uveitis, macular telangiectasia type 2 and hereditary retinal dystrophies.

The study was approved by the ethics committee of the University Hospital Jena and adhered to the tenets of the Declaration of Helsinki. All participants gave written informed consent prior to inclusion in the study and underwent a comprehensive ophthalmologic examination, which included best‐corrected visual acuity, OCT (Cirrus‐OCT 5000, Carl‐Zeiss Meditec AG, Jena, Germany, macula cube: 512 A‐scans per 128 B‐scans, axial resolution: 5 μm, lateral resolution: 15 μm). Pupils were dilated with tropicamide (Mydriaticum Stulln, Pharma Stulln GmbH, Nabburg, Germany) and phenylephrine hydrochloride (Neosynephrin‐POS 5%, Ursapharm GmbH, Saarbrucken, Germany). Following pupil dilation, patients underwent FLIO imaging. No sodium fluorescein was administered to the cornea or via intravenous injection prior to the FLIO investigation.

### 2.2 FLIO imaging and data analysis

The basic principles and laser safety of FLIO are described in detail elsewhere (Dysli et al., 2014; Sauer et al., 2015; Schweitzer et al., 2004). The recording of FLIO images utilises 473 nm picosecond laser diode excitation (repetition rate of 80 MHz), coupled with a laser scanning ophthalmoscope (Spectralis, Heidelberg Engineering, Heidelberg, Germany). Fluorescence photons were detected using time‐correlated single photon counting (SPC‐150, Becker & Hickl GmbH, Berlin, Germany) across two spectral channels: a short‐wavelength (SSC: 498–560 nm) and a long‐wavelength (LSC: 560–720 nm) channel. FLIO captures 30° field images with a frame rate of nine frames per second and a resolution of 256 × 256 pixels. Photon histograms over time for each pixel, which describe the autofluorescence decay, were fitted with a series of three‐exponential functions, using a least square method, in the software SPCImage 6.0 (Becker & Hickl GmbH, Berlin, Germany). The amplitude‐weighted mean decay time *τ*_m_, called FAF lifetime, was utilised for further analysis. The resulting image is colour‐coded, with short lifetimes represented in red and long lifetimes in blue. Additionally, the peak emission wavelength (PEW) of the fluorescence was determined from the ratio of photon counts (autofluorescence intensity) in SSC and LSC as described by Schultz, Hasan, Curcio, et al. (2021), Schultz, Hasan, Schwanengel, et al. (2021), Schultz, Klemm, et al. (2021), Schultz, Schwanengel, et al. (2021).

A standard ETDRS grid was centred on the fovea using the software FLIMX, which is documented and freely available for download under an open‐source Berkeley Software Distribution (BSD) licence (http://www.flimx.de) (Klemm et al., 2015). Mean FAF lifetimes and PEW per pixel were averaged across all pixels in the central circle, as well as in the inner and outer rings of the grid.

### 2.3 Statistics

SPSS version 27.0 (IBM, SPSS Inc., Chicago, IL, USA) was utilized for statistical analysis. Since not all data in all groups followed a normal distribution (as determined by the Kolmogorov–Smirnov test), median values of FAF lifetimes and PEW for all ETDRS‐grid areas were compared between AMD patients and controls using the Mann–Whitney *U*‐test. In subgroup analyses, the parameters of AMD patients with and without subretinal drusenoid deposits (SDD) were compared, and the correlation of FAF lifetimes and PEW with the subjects' ages was assessed. The significance level was set at 0.05.

## 3 RESULTS

Forty‐four AMD patients (mean age 80.0 ± 6.0 years) and 26 control subjects (mean age 73.0 ± 10.2 years) were included. As the mean ages between the two groups were different, we selected an age‐matched subgroup of 25 patients (mean age 76.3 ± 5.6 years) for the comparison of FAF lifetimes and PEW with those of the controls, excluding subjects older than 82 years. By contrast, for analysis of data within the patient group, all 44 AMD patients were included.

**FIGURE 1:**
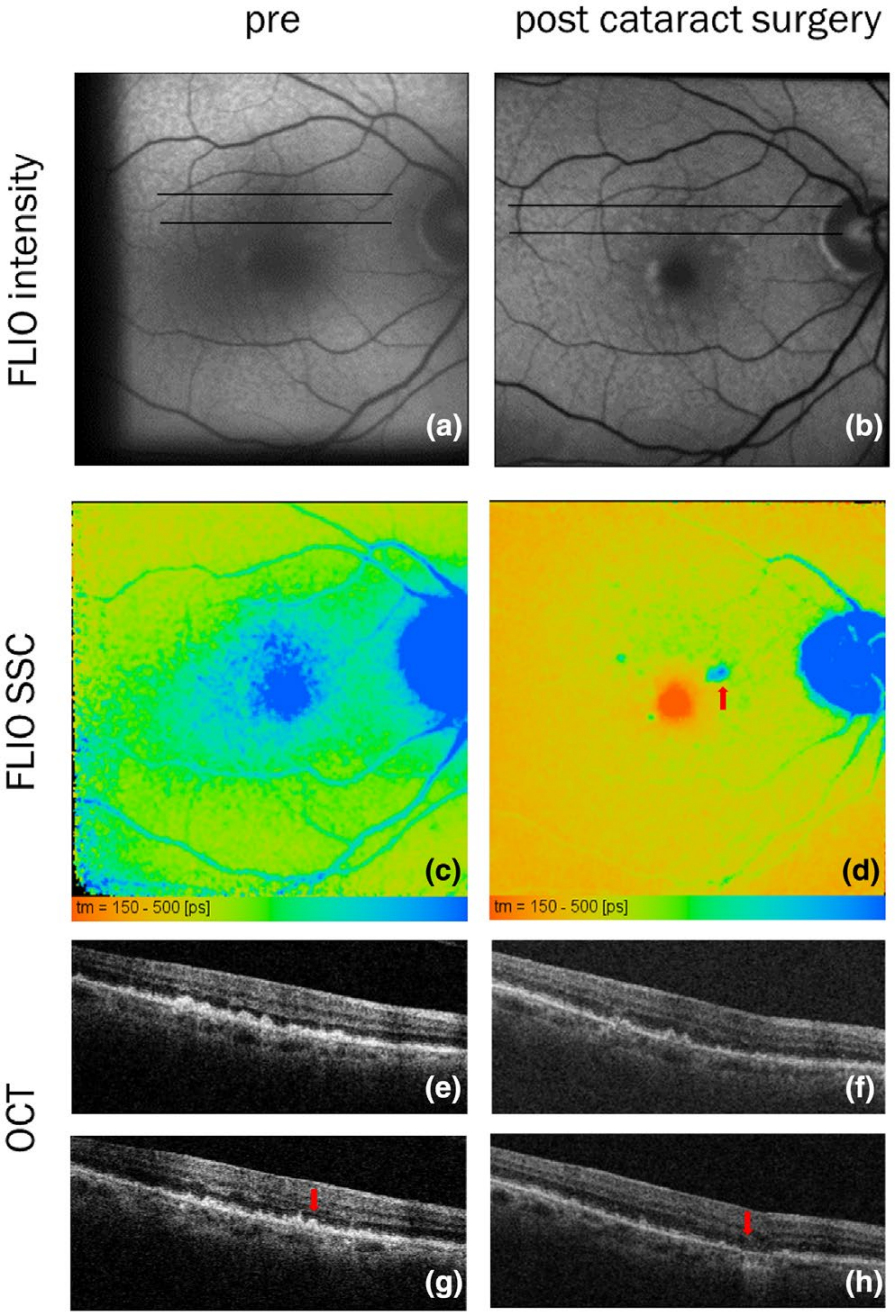
Seventy‐one‐year‐old patient before (left) and after (right) cataract extraction. Fluorescence intensity (a and b) and FLIO in SSC (c and d). OCT sections at black lines in a and b (e–h). The red arrow points to a druse topped with pathologically activated RPE turning into atrophy at follow‐up.

It is well established that lens fluorescence affects the measurement of retinal fluorescence lifetimes (Brauer et al., 2020; Schweitzer et al., 2020) and PEW (Simon, Brauer, et al., 2022; Simon, Curcio, et al., 2022) in healthy subjects. This effect has also been observed in AMD patients as illustrated in Figure 1. The figure depicts a 71‐year‐old female patient from whom we obtained FLIO measurements also prior to cataract extraction. She presented with intermediate AMD and exhibited drusen associated with activated, that is dysmorphic or migrating, RPE (Curcio et al., 2017) as well as SDD. The blue‐green colour in the pre‐surgery FLIO indicates longer FAF lifetimes compared with the post‐surgery image, which displays a red‐green coloration. This difference is particularly striking in the fovea, where the FAF intensity is low and, thus, the relative contribution of lens fluorescence is strongest. Furthermore, in this patient, AMD‐specific lesions are observed in OCT before and after cataract extraction, whereas they are only visible in FLIO in pseudophakia.

**FIGURE 2:**
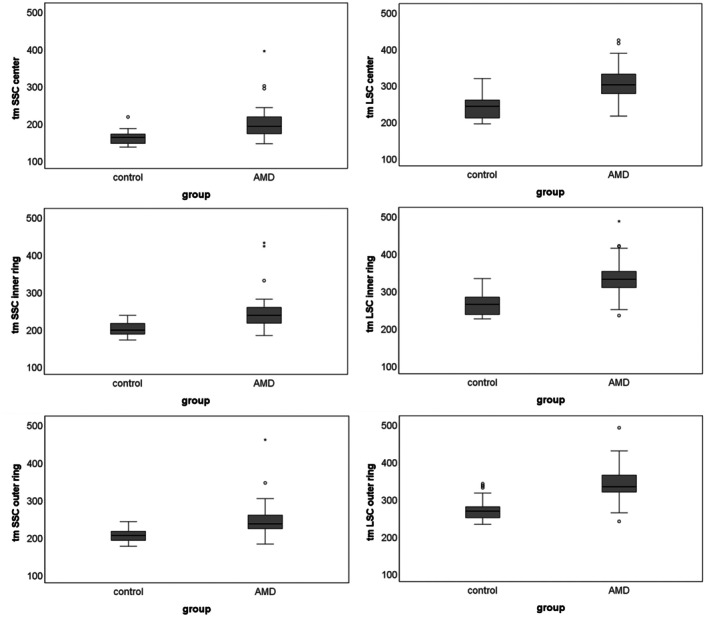
Boxplots of fluorescence lifetimes in controls and age‐matched AMD patients.

The median fluorescence lifetimes and PEW are presented in Table 1. The age‐matched subgroup of AMD patients (*N* = 25) showed significantly longer lifetimes compared with the control group across all areas of the ETDRS grid (all *p* < 0.001, Figure 2). The peripapillary lengthening of FAF lifetimes, described as a sign of AMD (Sauer, Andersen, et al., 2018; Sauer, Gensure, et al., 2018), was clearly seen in 24 patients, questionable in 15 patients and absent in 5 patients. The PEW were shorter in AMD than in controls (Table 1), with a significant difference observed only in the outer ring of the ETDRS grid (*p* = 0.039). A subgroup analysis of the AMD patients revealed that those with SDD had longer lifetimes than those without SDD (Table 2), although no significant difference in PEW was found.

**TABLE 1:** Median [interquartile range] of FAF lifetimes and PEW for controls, AMD patients and the subgroup of AMD patients, age‐matched to the controls. ‘centre’, ‘inner’ and ‘outer’ ring describe the respective fields of the ETDRS grid.

	*N*	*τ*_m_ SSC [ps]			*τ*_m_ LSC [ps]			PEW [nm]		
		Centre	Inner	Outer	Centre	Inner	Outer	Centre	Inner	Outer
Control	26	161 [26]	196 [28]	204 [26]	241 [49]	263 [48]	266 [34]	612 [21]	615 [14]	613 [20]
AMD	44	191 [39]	236 [36]	238 [40]	299 [53]	334 [46]	350 [62]	596 [26]	607 [20]	603 [16]
AMD age‐matched	25	191 [50]	236 [46]	235 [42]	300 [67]	330 [52]	332 [47]	600 [28]	607 [25]	603 [18]

**TABLE 2:** Median [interquartile range] of FAF lifetimes and PEW for AMD patients with and without SDD along with the *p*‐value from Mann–Whitney *U* test. ‘centre’, ‘inner’ and ‘outer’ ring describe the respective fields of the ETDRS grid.

	*N*	*τ*_m_ SSC [ps]			*τ*_m_ LSC [ps]			PEW [nm]		
		Centre	Inner	Outer	Centre	Inner	Outer	Centre	Inner	Outer
SDD	23	197 [44]	243 [36]	246 [34]	308 [48]	351 [34]	362 [54]	596 [18]	607 [18]	602 [15]
No DD	21	185 [40]	227 [38]	232 [42]	277 [45]	326 [48]	324 [75]	601 [34]	605 [26]	604 [22]
*p*‐Value		0.042	0.015	0.068	0.003	0.004	0.032	0.698	0.672	0.495

**FIGURE 3:**
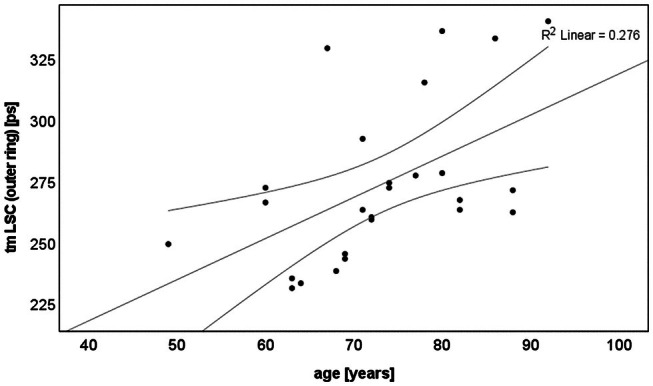
Age dependence of FAF lifetimes in LSC in the outer ETDRS‐grid ring for the controls (*p* = 0.006).

FAF lifetimes increased with age (*p* = 0.006–0.03) in the control group, except for that of SSC in the fovea (centre of the ETDRS grid). The strongest correlation was observed for LSC in both the inner and outer ETDRS rings (Figures 3 and S1). By contrast, no correlation was found in the patient group (Figure S2). Additionally, PEW showed no correlation with age in either the control or patient groups.

## 4 DISCUSSION

It is well established that the natural ocular lens contributes to the measurement of FAF. Since the lens has longer lifetimes than the fluorophores present in RPE and retina, this results in an apparent increase in FAF lifetimes and a decrease in PEW, particularly in elderly individuals and those with cataracts (Brauer et al., 2020; Lincke et al., 2021; Simon, Brauer, et al., 2022; Simon, Curcio, et al., 2022). This is illustrated in Figure 1. The influence of the lens is most pronounced in the fovea, where FAF is weak due to the absorption of excitation light by the macular pigment. Figure 1 (left) displays prolonged lifetimes in the fovea prior to cataract extraction, in contrast to the expected shorter lifetimes shown in Figure 1 (right) (Sauer et al., 2015; Sauer, Andersen, et al., 2018; Sauer, Gensure, et al., 2018). Additionally, longer FAF lifetimes observed at the sites of SDD and activated RPE, demonstrated by OCT and visible in FLIO after cataract surgery, are obscured by lens fluorescence in cataract‐affected eyes.

Generally, AMD is known to increase FAF lifetimes (Dysli et al., 2017; Sauer et al., 2019; Sauer, Andersen, et al., 2018; Sauer, Gensure, et al., 2018) and decrease PEW (Schultz, Hasan, Curcio, et al., 2021; Schultz, Hasan, Schwanengel, et al., 2021; Schultz, Klemm, et al., 2021; Schultz, Schwanengel, et al., 2021). In phakic patients, however, it was unclear which shifts in lifetime and PEW should be attributed to the lens, and how large the shift caused by the disease is. Although Dysli et al. (2017) found longer FAF lifetimes in pseudophakic AMD patients vs. controls, the number of pseudophakic subjects, particularly among the control group (*N* = 5), in this previous study was limited, making it challenging to conclude that lens fluorescence is not a significant confounding factor in FLIO in AMD. Here we found considerably smaller average differences in lifetimes between AMD patients and controls compared with Dysli et al. in the SSC (31 vs. 79 ps), but similar values in LSC (66 vs. 56 ps) (Dysli et al., 2017). That means that the influence of the lens might have been underestimated in this previous study for SSC, which is more affected by lens fluorescence. Nevertheless, our results demonstrate significantly longer FAF lifetimes in AMD patients compared with controls across all retinal areas. This confirms that AMD indeed increases FAF lifetimes. However, lens fluorescence must be considered a confounding factor in FLIO measurements in phakic AMD eyes. In two studies involving mixed phakic and pseudophakic non‐neovascular (Sauer et al., 2019; Sauer, Andersen, et al., 2018; Sauer, Gensure, et al., 2018) considerably longer FAF lifetimes in SSC (controls: 313/327 ps vs. 204 ps, AMD 342/388 ps vs. 238 ps) and moderately longer values in LSC (controls: 340/361 ps vs. 266 ps, AMD: 403/438 ps vs. 350 ps) were found. The stronger effect observed in the SSC was anticipated due to the relatively short‐wavelength fluorescence of the lens (Kessel et al., 2002; Kurzel et al., 1973; Zuclich et al., 2005). Therefore, the absolute values of FAF lifetimes, measured in phakic eyes, including those of AMD patients, are influenced by the long lifetimes of lens fluorescence and should be interpreted with caution. However, the ratio of FAF lifetimes between AMD patients and controls was similar across all spectral channels in both Sauer's studies and this study (1.09–1.28). Thus, the observed lengthening of FAF lifetimes in AMD, which has been reported in previous studies that also included phakic patients, is confirmed here and is not an artefact of lens fluorescence. The lens influence cannot be quantified from our measurements, but it can be estimated by comparing our data with that reported from a cohort of phakic subjects: Dysli et al. found the fluorescence lifetime in AMD to be longer than in control subjects by 267 ps in SSC and by 175 ps in LSC (Dysli et al., 2017), whereas we found that pseudophakic eyes exhibited longer lifetimes of only 31 ps and 66 ps, respectively. Although absolute FAF lifetimes may differ between both studies as we used three‐exponential decay fits in contrast to Dysli et al., who used two exponentials, the lifetime differences should not be greatly affected. This indicates a significant lens influence, specifically in SSC, which aligns with the finding that lens fluorescence contributes up to 55% in SSC and 25%–30% in LSC to the total measured fluorescence in cataract eyes (Brauer et al., 2020). Further investigations involving AMD patients, both before and after cataract extraction, should be conducted to quantitatively evaluate the influence of the lens on FAF lifetimes in AMD.

Little is known about the influence of the eye lens on FAF spectra. In this study, the peak emission wavelength (PEW) was only slightly shorter in patients compared with controls. By contrast, Schultz et al. reported significantly shorter PEW values in a mixed population of phakic and pseudophakic individuals (Schultz, Hasan, Curcio, et al., 2021; Schultz, Hasan, Schwanengel, et al., 2021; Schultz, Klemm, et al., 2021; Schultz, Schwanengel, et al., 2021). Additionally, another study indicates that cataract extraction leads to a significant increase in PEW (Simon, Brauer, et al., 2022; Simon, Curcio, et al., 2022). Therefore, the apparently shorter PEW in AMD patients in a previous study (Schultz, Hasan, Curcio, et al., 2021; Schultz, Hasan, Schwanengel, et al., 2021; Schultz, Klemm, et al., 2021; Schultz, Schwanengel, et al., 2021) may reflect a hypsochromic spectral shift due to lens fluorescence contributions. However, it is also important to consider the age differences between patients and controls in that study.

A previous study found significantly longer FAF lifetimes in the inner and outer ring of the ETDRS grid for AMD patients having SDD (Simon, Brauer, et al., 2022; Simon, Curcio, et al., 2022). This finding has been confirmed here. Moreover, in pseudophakic eyes, we observed significantly longer FAF lifetimes in patients with SDD also in the centre of the grid (see Table 2). This effect may have been obscured by the relatively strong influence of lens fluorescence in the fovea of phakic eyes in the previous study.

The association between FAF lifetime and age is well established in phakic healthy subjects (Dysli et al., 2014; Sauer et al., 2020). A similar correlation was observed in the pseudophakic healthy subjects in LSC but not in SSC (Dysli et al., 2023). In contrast to this study, we found a correlation of lifetimes with the control subjects' ages for both spectral channels (except for the fovea in SSC), suggesting that the increase in FAF lifetime is not merely a lens artefact, but not in AMD patients. However, it is important to consider that the age range of the patients was narrower than that of the controls, as AMD typically occurs at an older age. Additionally, the number of subjects was greater in the patient group compared with the control group (44 vs. 26). Since no correlation was identified despite the larger patient group, we hypothesize that the variability in FAF lifetimes associated with the disease may overshadow the effect of age. This highlights FLIO's capability to indicate changes in the retinal and RPE metabolic state, including the accumulation of lipids (Curcio, 2018; Curcio et al., 2001, 2011) and bis‐retinoids (Sparrow et al., 2012) as well as their oxidation products (Ben‐Shabat et al., 2002; Sparrow et al., 2002).

Despite the limitations posed by the small number of subjects, particularly within the control and age‐matched patient groups, this investigation supports earlier findings regarding the age dependence of FAF lifetimes and their prolongation in AMD patients, specifically, those with SDD. This is significant because previous studies have reported conflicting results regarding the influence of the lens on FAF lifetimes in AMD. While some investigations indicate significantly longer FAF lifetimes in phakic AMD patients compared with pseudophakic ones (Dysli et al., 2017; Sauer, Andersen, et al., 2018; Sauer, Gensure, et al., 2018), others have found no differences (Goerdt et al., 2021; Hammer et al., 2020) or have ruled out the lens's influence by reporting only local lifetime differences within the same eyes (Hammer et al., 2020, 2021; Schwanengel et al., 2022; Weber et al., 2022). This raised questions about whether the observed changes in FAF lifetimes genuinely reflect alterations in FAF or are artefacts resulting from lens fluorescence, which can vary with age and disease. This study confirms the elongation of FAF lifetimes in AMD independent of lens fluorescence.

## Supporting information


Data S1
