# Supplementary material for: Fundus autofluorescence lifetimes in age‐related macular degeneration versus healthy controls in a pseudophakic population
Source: Acta Ophthalmol. 2025 May 14;103(6):e394–400. doi: 10.1111/aos.17519 (PMC12340172; doi:10.1111/aos.17519)
Supplement: Supplementary file 1 — Data S1 [file AOS-103-e394-s001.docx]

Supplementary material

Figure S1: Age-dependence of FAF-lifetimes in LSC in the inner ETDRS-grid ring for the control subjects.


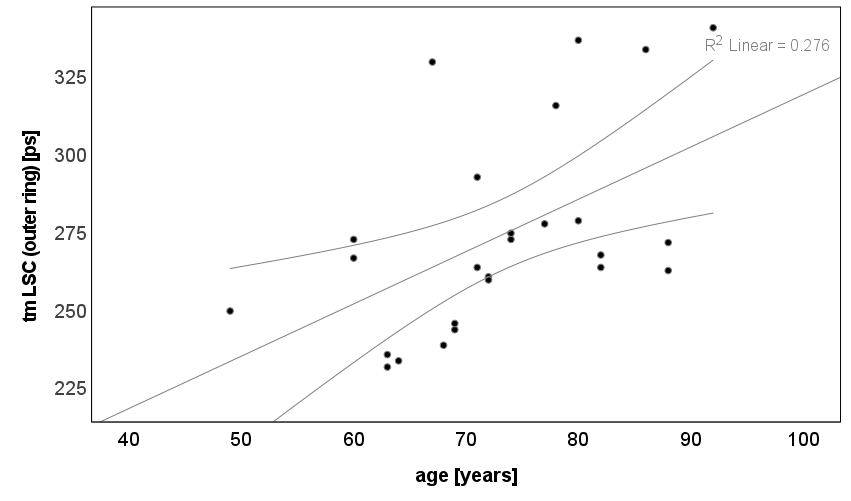


Figure S2: Age-dependence of FAF-lifetimes in LSC in the inner and outer ETDRS-grid ring for the AMD patients.


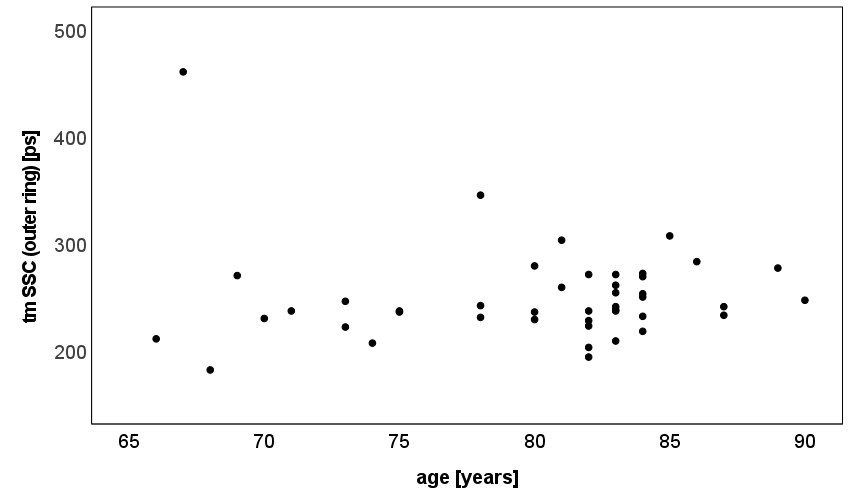

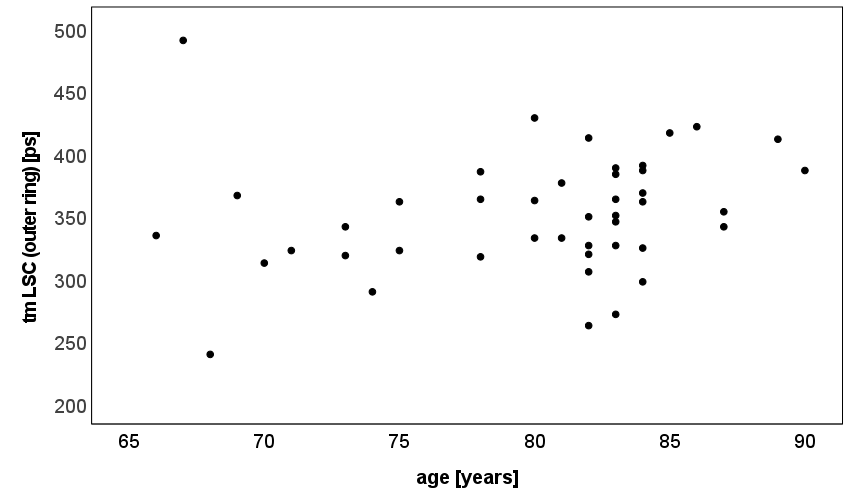


Table S1: Amplitudes a_1-3_ and lifetimes τ_1−3_ (fixed per image according to Ref. 27) from SSC for patients and controls in the ETDRS grid segments (mean±SD)

|  | ETDRS-grid | a_1_ [%] | a_2_ [%] | a_3_ [%] | τ_1_ [ps] | τ_2_ [ps] | τ_3_ [ps] |
| --- | --- | --- | --- | --- | --- | --- | --- |
| control | center | 95.8±2.7 | 3.1±2.6 | 1.1±0.3 | 101.8±5.2 | 615.4±15.4 | 4160.8±192.0 |
|  | inner ring | 89.1±2.6 | 9.6±2.5 | 1.3±0.3 |  |  |  |
|  | outer ring | 86.7±2.3 | 12.2±2.2 | 1.1±0.3 |  |  |  |
| AMD | center | 91.5±2.8 | 7.6±2.6 | 0.9±0.3 | 107.3±2.6 | 632.1±7.8 | 4366.7±95.8 |
|  | inner ring | 84.5±2.8 | 14.3±2.5 | 1.2±0.4 |  |  |  |
|  | outer ring | 82.7±3.4 | 16.3±3.2 | 1.1±0.3 |  |  |  |

Table S2: Amplitudes a_1-3_ and lifetimes τ_1−3_ (fixed per image according Ref. 27^1^) from LSC for patients and controls in the ETDRS grid segments (mean±SD)

|  | ETDRS-grid | a_1_ [%] | a_2_ [%] | a_3_ [%] | τ_1_ [ps] | τ_2_ [ps] | τ_3_ [ps] |
| --- | --- | --- | --- | --- | --- | --- | --- |
| control | center | 89.5±4.0 | 8.3±3.6 | 2.2±0.5 | 135.1±6.8 | 653.4±23.6 | 3056.1±113.6 |
|  | inner ring | 83.1±3.4 | 14.8±3.1 | 2.0±0.5 |  |  |  |
|  | outer ring | 81.1±3.1 | 17.2±2.7 | 1.8±0.5 |  |  |  |
| AMD | center | 82.6±3.9 | 15.3±3.6 | 2.1±0.6 | 142.3±3.4 | 678.8±11.7 | 3178.5±56.2 |
|  | inner ring | 75.4±4.3 | 22.1±3.7 | 2.4±0.7 |  |  |  |
|  | outer ring | 72.8±5.2 | 24.9±4.6 | 2.3±0.7 |  |  |  |
